# The Association Between Early Antenatal Care and Intermittent Preventive Treatment of Malaria in Pregnancy in Sub-Saharan Africa: Effect Modification by Planned Pregnancy Status

**DOI:** 10.5334/aogh.3550

**Published:** 2022-01-10

**Authors:** Paschal Awingura Apanga, Maxwell Tii Kumbeni, Mary-Ann Wepiamo Chanase

**Affiliations:** 1 School of Public Health, University of Nevada, Reno, Reno, Nevada, US; 2 Oregon State University, College of Public Health and Human Sciences, Corvallis, Oregon, US; 3 University of Illinois, College of Applied Health Science, Urbana-Champaign, US

## Abstract

**Background:**

Evidence of the association between early antenatal care (ANC) and receiving at least three doses of sulphadoxine–pyrimethamine (IPTp3+) during pregnancy is limited. It’s also unclear whether the association between early ANC and IPTp3+ is modified by planned pregnancy status.

**Objectives:**

Our primary aim was to assess the relationship between early ANC and IPTp3+ and to assess whether this relationship is modified by a woman’s planned pregnancy status. We also estimated IPTp3+ coverage across Sub-Saharan African countries.

**Methods:**

Data on 77 183 mothers with a live birth in the past two years were analyzed using multiple indicator cluster surveys (MICSs) from 17 Sub-Saharan African countries conducted between 2013 and 2019. We used modified Poisson regression with a robust variance to assess the association between early ANC and IPTp3+, while adjusting for country, clustering, stratification and sample weights. Effect modification by planned pregnancy status was assessed on the additive and multiplicative scales. We used meta-analytic techniques to pool prevalent estimates of IPTp3+ across all countries.

**Findings:**

IPTp3+ overall coverage was 22.1% (95% CI: 17.0%, 27.1%), and ranged from 2.9% (95% CI: 1.3%, 4.4%) in São Tomé and Príncipe to 51.7% (95% CI: 49.2%, 54.1%) in Ghana. IPTp3+ coverage was 30% higher among mothers who had early ANC compared to those who did not have early ANC [adjusted prevalence ratio (aPR): 1.30, 95% CI: 1.23,1.36]. There was evidence of effect modification on the additive [relative excess risk due to interaction (RERI): 0.08, 95% CI: 0.0002, 0.15] and multiplicative (aPR: 1.10, 95% CI: 1.01, 1.20) scales.

**Conclusions:**

IPTp3+ coverage was low across many of the countries in Sub-Saharan Africa. Women who had early ANC were more likely to receive IPTp3+. Women whose pregnancies were unplanned were less likely to receive IPTp3+, but our effect modification analysis showed that early ANC among such women can increase IPTp3+ coverage.

## Introduction

An estimated 33 million pregnancies were exposed to malaria infection within the Sub-Saharan African region in 2019 [1]. Malaria in pregnancy is a major preventable public health problem with substantial risk to the mother, foetus and newborn [2,3]. It is associated with maternal and infant morbidity and mortality, including adverse birth outcomes such as low birthweight, stillbirths, and neonatal deaths [3,4,5]. The World Health Organization (WHO) recommends a three-prong approach in the prevention and treatment of malaria in pregnancy, which includes intermittent preventive treatment in pregnancy (IPTp) with sulphadoxine–pyrimethamine (SP), the use of insecticide treated nets and effective case management [1]. In 2012, the WHO updated its guidelines on IPTp with SP from a minimum of two doses of SP (IPTp2+) to at least three doses of SP (IPTp3+) [6,7], based on evidence that IPTp3+ was associated with higher mean birthweight, fewer low birthweights and less placental malaria [8]. The WHO advocates for pregnant women living in settings of moderate to high malaria transmission receive IPTp3+ [6]. It is recommended that IPTp with SP should be administered as early as possible within the second trimester of pregnancy and subsequent doses of SP given at each scheduled antenatal care (ANC) contact with an interval of one month apart until delivery [6]. Despite these recommendations, only 34% received IPTp3+ in 2019, which remains well below the target of 80% [1].

Early ANC is the first ANC contact by the pregnant woman to the healthcare provider within the first trimester of pregnancy [9]. Although IPTp with SP is contraindicated within the first trimester of pregnancy [6], early ANC provides skilled healthcare workers the opportunity to discuss ANC schedules as well as educate pregnant woman on the importance of ANC, including IPTp with SP [10]. Early ANC also provides the opportunity for healthcare workers to screen/investigate pregnant women for diseases as well as get to understand any pre-existing health condition the woman might have [11]. Due to the significance of early ANC, it may play an important role in increasing the coverage of IPTp3+. However, there are limited studies on the relationship between early ANC and IPTp3+ [12,13,14]. In addition, no multicountry study has assessed the relationship between early ANC and IPTp3+ across the Sub-Saharan African region. It is also unknown whether the relationship between early ANC and IPTp3+ is modified by a woman’s planned pregnancy status.

Our objective in this study was to estimate the relationship between early ANC and IPTp3+, and to assess whether this relationship is modified by a woman’s planned pregnancy status. We hypothesized that women who had early ANC are more likely to receive IPTp3+, and that this relationship will be modified by whether her pregnancy was planned or not. We also assessed IPTp3+ coverage across 17 Sub-Saharan African countries using data from multiple indicator cluster surveys (MICS) collected after the WHO 2012 recommendations on IPTp3+.

## Methods

### Study design, setting and data collection

We used data from the MICS from 17 Sub-Saharan African countries collected between 2013 and 2019. The MICS are cross-sectional, population-based cluster surveys based on nationally representative household samples [15]. The study population was mothers with a live birth in the past two years living in the following Sub-Saharan African countries: Benin, Central African Republic (CAR), Cameroon, Congo, Democratic Republic of Congo (DRC), Gambia, Ghana, Guinea, Guinea Bissau, Ivory Coast, Madagascar, Malawi, Mali, Mauritania, Nigeria, São Tomé and Príncipe (STP) and Togo.

The MICSs use a two-stage sampling technique in each country. In the first stage, enumeration areas are drawn from census files, and in the second stage, households are randomly selected from a list of households in each enumeration area. Household participation rates are usually 90–95% [15]. The MICS sampling design and procedures have been previously described in detail [16,17].

### Exposures

The exposure was early ANC, which was defined as mothers who had ANC contact with the healthcare provider within the first trimester of pregnancy [9]. Early ANC was assigned “1” to mothers who had early ANC and “0” to those who did not. Planned pregnancy status was assessed as an effect modifier and was dichotomized as either unplanned or planned pregnancy.

### Outcome measure

Our primary outcome of interest was IPTp3+. The outcome measure was dichotomized as “1” for mothers who received three or more doses of SP during pregnancy and “0” for mothers who received less than three doses of SP during pregnancy.

### Covariates

The covariates in our study included: age, marital status, educational level of mothers, household wealth, residential status, parity, access to media and perceived domestic violence. Covariates were categorized as: marital status (married/cohabitation, single); educational level of mothers (≥ secondary education, < secondary education); residential status (urban, rural); and parity (multiparous, primiparous). Age was assessed as a continuous variable and household wealth was categorized into wealth quintiles (poorest, poor, middle, rich and richest). Household wealth was assessed as a composite indicator of wealth derived from principal component analysis using household assets [16]. Access to media (yes, no) was defined as having access to any of the following: reading the newspaper/magazine, listening to radio, watching television or internet services [18]. Perceived domestic violence was also dichotomized as either perceived domestic violence or no perceived domestic violence. Domestic violence was considered perceived if a mother reported that her husband/partner was not justified in beating/hitting her if she committed any of the following events: if she goes out without telling him; if she neglects the children; if she argues with him; if she refuses to have sex with him, and if she burns the food [18].

### Statistical analyzes

We used descriptive statistics to assess the study population stratified by each country. Categorical variables were presented as percentages, while the continuous variable was presented as mean and standard deviation.

Descriptive statistics was also used to assess the prevalence of intermittent preventive treatment with at least one dose (IPTp1+), IPTp2+ and IPTp3+ stratified by each country. Random-effects meta-analysis with inverse variance weighting was used to pool prevalent estimates of our results on intermittent preventive treatment across all countries. Statistical heterogeneity was reported using the I^2^ statistics. While an I^2^ > 50% may be of substantial heterogeneity, an I^2^ > 75% may be of considerable heterogeneity [19]. Our results are presented by each country and overall using forest plots.

We used two separate modified Poisson regressions with robust error variance to conduct our regression analyzes. To ensure the representativeness of our data, each of these models followed MICS guidelines [20], and adjusted for the sample design, taking into account country, sampling weight, clustering, and stratification variables.

The first model assessed the relationship between early ANC and IPTp3+, while adjusting for potential confounders, which include planned pregnancy status, age, marital status, educational level of mothers, household wealth, residential status, parity, access to media and perceived domestic violence. Potential confounders were specified as *a priori*, as we thought there is a biological plausibility that these variables were associated with both the exposure and outcome of interest. The second model was similar to the first, but included an interaction term between early ANC and planned pregnancy status (early ANC*planned pregnancy status). Effect modification was assessed on the additive and multiplicative scale, while adjusting for the same variables as in the first model.

Effect modification on the additive scale was assessed using the relative excess risk due to interaction (RERI) as this is the most appropriate public health measure for assessing effect modification on the additive scale [21,22]. We used publicly available SAS codes to estimate the RERI and corresponding 95% confidence intervals [23]. We present our effect modification results in a tabular format recommended by Knol and VanderWeele [24].

The variables for our analyzes were pre-selected based on factors identified in previous literature, [14,25,26] and availability in the MICS datasets. While we used Stata 16 SE (Stata Corp, College Station, TX) to conduct the meta-analysis, SAS version 9.4 (SAS Institute, Cary, NC) was used for the descriptive statistics and regression analyzes. Missing data were excluded from the study. The proportion of individuals with missing information in our study was 1%.

### Sensitivity analyzes

We conducted sensitivity analyzes to determine whether our findings were sensitive to different levels of our outcome. We assessed whether the relationship between early ANC and the prevalence of IPTp1+ was modified by planned pregnancy status. In addition, we determined whether planned pregnancy status modified the association between early ANC and the prevalence of IPTp2+.

### Ethics

This study does not require ethical approval because we used deidentified secondary data, which is publicly available.

## Results

### Descriptive statistics

A total of 77183 mothers with a live birth in the past two years were included in the study (***Table 1***). The flow diagram for screening and selection of MICS datasets for this analysis is shown in Supplementary Figure S1. A total of 343 surveys conducted between January 1993 to December 2020 were screened for inclusion into our study. We excluded surveys that were conducted before the WHO 2012 updated recommendations on IPTp3+ [6]. This study included only countries within the African region as there is currently insufficient evidence to support a general recommendation of IPTp with SP use outside Africa [6]. We also excluded countries that were certified malaria-free by WHO [27], and countries that did not have available data. Countries with data limited to only select counties/regions were also excluded (Supplementary Figure S1). This study identified 17 surveys conducted between January 2013 and December 2019.

**Table 1:** Characteristics of the study population across 17 Sub-Saharan African countries (n = 77183).


COUNTRY	BENIN	CAR	CAMEROON	CONGO	DRC	GAMBIA	GHANA	GUINEA	GUINEA BISSAU	IVORY COAST	MADAGASCAR	MALAWI	MALI	MAURITANIA	NIGERIA	STP	TOGO

**Survey year**	2014	2018–2019	2014	2014–2015	2017–2018	2018	2017–2018	2016	2018–2019	2016	2018	2013–2014	2015	2015	2016–2017	2019	2017

**Sample size**	5388	3565	2977	2956	7648	3472	3529	3879	2860	3390	4940	7490	6756	4150	11547	664	1971

**Age; mean (SD)**	28 (6.7)	27 (7.0)	27 (6.7)	28 (6.1)	29 (6.6)	29 (6.2)	29 (7.1)	28 (7.0)	28 (6.8)	27 (6.3)	27 (7.1)	27 (6.7)	28 (7.0)	29 (7.1)	29 (7.1)	28 (6.8)	29 (6.7)

**Marital status; (%)**																	

Single	5.5	13.8	17.9	19.5	15.1	6.2	17	5	15.6	17.5	19.3	15.4	3.7	8.1	3.9	25	7.9

Married/cohabitation	94.5	86.2	82.1	80.5	84.9	93.8	83	95	84.4	82.5	80.7	84.6	96.3	91.9	96.1	75	92.1

Missing			3			2									10		2

**Education; (%)**																	

<Secondary education	86	95	64.5	33.4	52	66.2	43.4	84.6	91.4	83.2	69.9	82.6	94.5	82.4	64.8	43.9	69.9

≥Secondary education	14	5	35.5	66.6	48	33.8	56.6	15.4	8.6	16.8	30.1	17.4	5.5	17.6	35.2	56.1	30.1

Missing					5								1	15		1	

**Household’s wealth; (%)**																	

Poorest quintile	20.3	22.2	23.6	24.3	24.3	22.8	21.6	20.7	21.2	25.2	25.2	24.7	19.7	21.1	22.4	24.6	20.9

Poor quintile	20.9	22.4	22.1	22.4	21.6	21.8	20	22.8	23.6	21.8	22.6	22.4	20.7	21	22.1	20.5	21.4

Middle quintile	20.3	21.1	21.1	20.3	19.7	20.4	19.5	20.5	22.4	20.4	19.7	20.8	20.9	19.9	19.7	18.1	19.6

Rich quintile	21	18.6	18	17.8	19.5	18.8	20.4	20	18.5	18	17.2	16.6	21.2	20.1	18.3	21.3	20.6

Richest quintile	17.4	15.7	15.2	15.2	14.9	16.2	18.5	16	14.4	14.5	15.4	15.5	17.5	18	17.6	15.5	17.5

**Area of residence; (%)**																	

Rural	56.7	69.5	59.3	38.4	61.5	37.8	57.7	66.5	73.2	60	80	88.1	80.9	55	70.3	34.3	60.4

Urban	43.3	30.5	40.7	61.6	38.5	62.2	42.3	33.5	26.8	40	20	11.9	19.1	45	29.7	65.7	39.6

**Planned pregnancy status; (%)**																	

Planned	62.7	48.7	77.1	56.7	59.8	74.7	49.4	74.9	84.1	62.4	86.3	54.8	76.1	74.3	79.1	33	56.7

Unplanned	37.3	51.3	22.9	43.3	40.2	25.3	50.6	25.1	15.9	37.6	13.7	45.2	23.9	25.7	20.9	67	43.3

Missing	1	27	48	5	10	9		8		1	10	75	44	26	12	4	

**Parity; (%)**																	

Primiparous	18.8	17.6	21.8	22.9	17.4	20.4	22.8	19.6	22.7	24.5	28.4	22.6	17.6	18.4	16.3	24.4	20.9

Multiparous	81.2	82.4	78.2	77.1	82.6	79.6	77.2	80.4	77.3	75.5	71.6	77.4	82.4	81.6	83.7	75.6	79.1

**Early ANC; (%)**																	

No	48.5	65.2	65.5	50.4	83.1	54.6	38.2	55.5	56.9	65.9	72.7	79.2	67.2	35.6	81.2	10.1	66.4

Yes	51.5	34.8	34.5	49.6	16.9	45.4	61.8	44.5	43.1	34.1	27.3	20.8	32.8	64.4	18.8	89.9	33.6

Missing		31			6	5			1		10		12			7	1

**Access to media; (%)**																	

No	2.1	72.2	4	5.2	64	7.8	15.1	1.4	3	2	47.3	6.7	0.4	5.2	3	4	30.4

Yes	97.9	27.8	96	94.8	36	92.2	84.9	98.6	97	98	52.7	93.3	99.6	94.8	97	96	69.6

Missing		6			64	3					1					1	

**Perceived domestic violence; (%)**																	

No	59.6	31.8	59.8	42.7	34.9	41	65.9	23.1	60.4	51.9	60.2	86.1	21.8	69.4	61.6	83.5	69.4

Yes	40.4	68.2	40.2	57.3	65.1	59	34.1	76.9	39.6	48.1	39.8	13.9	78.2	30.6	38.4	16.5	30.6

Missing		80			38	24	20				20					9	20


CAR: Central African Republic; DRC: Democratic Republic of the Congo; STP: São Tomé and Príncipe; SD: Standard deviation.

The mean age of mothers across each of the countries ranged from 27–29 years. The prevalence of early ANC ranged from 16.9% in DRC to 89.9% in STP. Majority of mothers had their pregnancies planned in 14 of the countries, except in CAR, Ghana and STP. Study participants came from a mix of rural and urban areas across each of the countries (***Table 1***).

### Prevalence of IPT with SP during pregnancy

**Figure 1:**
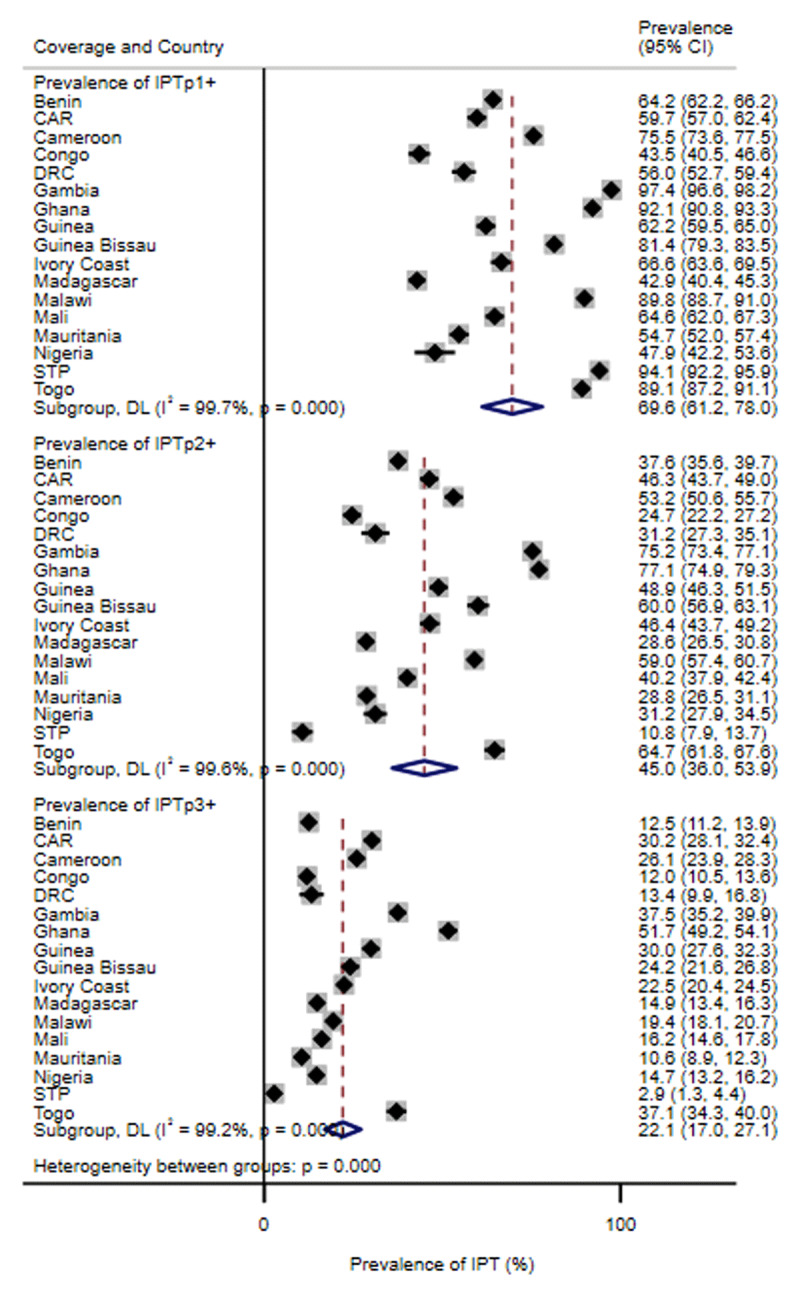
Coverage of intermittent preventive treatment of malaria in pregnancy (IPTp) with sulphadoxine–pyrimethamine (SP) by country stratified by the number of SP doses. CAR: Central African Republic; DRC: Democratic Republic of the Congo; STP: São Tomé and Príncipe. IPTp3+: three or more intermittent preventive treatment doses. IPTp2+: two or more intermittent preventive treatment doses. IPTp1+: one or more intermittent preventive treatment doses.

The meta-analysis showed the overall prevalence of IPTp3+ was 22.1% (95% CI: 17.0%, 27.1%) with substantial heterogeneity in the estimates across all countries (I^2^=99.2%, P-value < 0.0001; ***Figure 1***). All countries, except Ghana reported IPTp3+ prevalence less than 50%. The prevalence of IPTp3+ ranged from 2.9% (95% CI: 1.3%, 4.4%) in STP to 51.7% (95% CI: 49.2%, 54.1%) in Ghana. Not surprisingly, the prevalence of IPTp1+ and IPTp2+ were higher across all countries compared to IPTp3+ (***Figure 1***, Supplementary Table S1).

### Association between early ANC and IPTp3+

Among all mothers, the prevalence of IPTp3+ was 30% higher among those who had early ANC compared to mothers who did not have early ANC [adjusted prevalence ratio (aPR): 1.30, 95% CI: 1.23,1.36; ***Table 2***]. A number of factors were also associated with IPTp3+. The prevalence of IPTp3+ was 8% lower among mothers whose pregnancies were unplanned compared to those whose pregnancies were planned (aPR: 0.92, 95% CI: 0.88, 0.96). Mothers in the richest, rich, middle, and poor wealth quintiles were 1.11, 1.13, 1.13 and 1.08 times more likely to have received IPTp3+, respectively, compared to mothers in the poorest wealth quintile. The prevalence of IPTp3+ was 25% higher among mothers who had access to media compared to mothers who had no access to media (aPR: 1.25, 95% CI: 1.12, 1.39). Mothers who had perceived domestic violence had 10% lower prevalence of IPTp3+ compared to mothers who did not report such perception (aPR: 0.90, 95% CI: 0.84, 0.96; ***Table 2***).

**Table 2:** The association between early ANC and IPTp3+.


CHARACTERISTICS	ADJUSTED PR (95% CI)

**Early ANC**	

No	1.00 (Reference)

Yes	1.30 (1.23,1.36)

**Planned pregnancy status**	

Planned	1.00 (Reference)

Unplanned	0.92 (0.88,0.96)

Age	1.00 (1.00,1.00)

**Marital status**	

Single	1.00 (Reference)

Married/cohabitation	0.99 (0.93,1.06)

**Education**	

<Secondary education	1.00 (Reference)

≥Secondary education	0.99 (0.94,1.04)

**Household’s wealth**	

Poorest quintile	1.00 (Reference)

Poor quintile	1.08 (1.02,1.15)

Middle quintile	1.13 (1.04,1.22)

Rich quintile	1.13 (1.04,1.23)

Richest quintile	1.11 (1.01,1.23)

**Area of residence**	

Rural	1.00 (Reference)

Urban	1.04 (0.97,1.11)

**Parity**	

Primiparous	1.00 (Reference)

Multiparous	0.96 (0.91,1.01)

**Access to media**	

No	1.00 (Reference)

Yes	1.25 (1.12,1.39)

**Perceived domestic violence**	

No	1.00 (Reference)

Yes	0.90 (0.84,0.96)


PR: Prevalence ratio; ANC: antenatal care; IPTp3+: three or more intermittent preventive treatment doses.

### Effect modification by planned pregnancy status

We found that the relationship between early ANC and IPTp3+ was modified by planned pregnancy status on both multiplicative (aPR: 1.10, 95% CI: 1.01, 1.20), and additive (RERI: 0.08, 95% CI: 0.0002, 0.15), scales. Among mothers who did not have early ANC, those whose pregnancies were unplanned had 12% lower prevalence of receiving IPTp3+ compared to mothers whose pregnancies were planned. However, among mothers whose pregnancies were unplanned, the prevalence of IPTp3+ was 38% higher among those who had early ANC compared to mothers who did not have an early ANC (aPR: 1.38, 95% CI: 1.29, 1.48; ***Table 3***).

**Table 3:** Effect modification of the association between early antenatal care and IPTp3+ by planned pregnancy status.


	NO EARLY ANC		EARLY ANC		

	N with/without outcome	PR (95% CI)	N with/without outcome	PR (95% CI)	PR (95% CI) comparing IPTp3+ coverage with early ANC vs. not within strata of planned pregnancy status

Planned pregnancy	5093/23746	1.00 (Reference)	4755/14098	1.26 (1.18,1.34) p *< 0.01*	1.26 (1.18,1.34)p *< 0.01*

Unplanned pregnancy	2467/12231	0.88 (0.83,0.94) *p < 0.01*	2108/6374	1.22 (1.14,1.30) *p < 0.01*	1.38 (1.29,1.48)*p < 0.01*


Measure of effect modification on additive scale: RERI (95% CI) = 0.08 (0.0002, 0.15); p = 0.04. Measure of effect modification on multiplicative scale: ratio of PRs (95% CI) = 1.10 (1.01, 1.20); p = 0.04. Prevalence ratios (PRs) are adjusted for age, marital status, education, household wealth, place of residence, parity, access to media, perceived domestic violence. IPTp3+: three or more intermittent preventive treatment doses.

### Sensitivity analyzes

Our effect modification results were similar to different definitions of our outcome (Supplementary Table S2, Supplementary Table S3). Planned pregnancy status modified the association between early ANC and the prevalence of IPTp2+ (i.e., previous IPT recommendation with SP) on both the multiplicative and additive scales (Supplementary Table S2). Similarly, we found that the relationship between early ANC and the prevalence of IPTp1+ was modified by planned pregnancy status on the multiplicative and additive scales (Supplementary Table S3).

## Discussion

Our primary aim was to assess the relationship between early ANC and IPTp3+ and to assess whether this relationship is modified by planned pregnancy status. We also assessed the coverage of IPTp3+ across Sub-Saharan Africa. Our meta-analysis showed the overall prevalence of IPTp3+ was low and also low across many of the countries in Sub-Saharan Africa. Mothers who had early ANC were more likely to receive IPTp3+ compared to mothers who did not initiate early ANC. We also found evidence of positive effect modification by planned pregnancy status on the relationship between early ANC and IPTp3+ on both the additive and multiplicative scales. While the prevalence of IPTp3+ was low among all mothers whose pregnancies were unplanned, early ANC among such mothers was associated with a higher prevalence of IPTp3+.

The low coverage of IPTp+3 across many of the countries in our study could be due to several reasons. Some countries may not have adopted the updated WHO 2012 guidelines on IPTp+3 [28]. Another possible reason is that some pregnant women may not have access to ANC services [29]. In addition, some women did not receive IPTp+3 because they did not make the required ANC contacts needed to receive IPTp+3 even when services were available [30,31]. Stock-outs of SP has also been reported as a barrier to uptake of IPT with SP [32].

Our finding on the association between early ANC and IPTp+3 has been reported among women in Ghana [13], Malawi [12], and Bangladesh [14]. A number of reasons may account for our study findings across Sub-Saharan African countries. Pregnant women who had early ANC are educated on the importance of ANC including IPT with SP, which is given in the subsequent ANC schedule [6,10]. Such women are likely to return for their second ANC contact during which they receive their first dose of SP, which is associated with IPTp+3 [13]. Pregnant women who had early ANC may also already be aware of the significance of ANC and SP, and are more likely to make the required ANC contacts needed to receive IPTp+3.

While we found that among all mothers, women whose pregnancies were unplanned were less likely to receive IPTp+3 compared to mothers whose pregnancies were planned, our effect modification analysis showed that initiation of early ANC among women with unplanned pregnancies was associated with a higher coverage of IPTp+3 compared to pregnant women whose pregnancies were planned. This finding is unique and an important contribution to literature as public health programmes or interventions aimed at increasing the coverage of IPTp+3 may need to target pregnant women, particularly those whose pregnancies were unplanned to enable them initiate early ANC.

Our finding on the lower prevalence of IPTp+3 among mothers who had perceived domestic violence was not surprising. Previous studies on domestic violence were negatively associated with ANC [26,33], and therefore women who perceive/experience domestic violence may not meet the required ANC contacts needed to receive IPTp+3. Our finding on the association between mothers within higher wealth quintiles more likely to receive IPTp+3 compared to those in the poorest wealth quintile aligns well with published literature [34]. Our analysis also showed that mothers who had access to the media were more likely to receive IPTp+3 compared to those who did not, and this has previously been reported [25].

This study had several strengths and limitations. This study employed a large cross-sectional data with a data collection, which is designed to be standardized, rigorous and internationally comparable across each of the countries in our study [16]. The MICSs are nationally representative household surveys, and therefore our findings should be generalizable to the countries in our study. A major limitation is that our findings cannot be interpreted causally as this was a cross-sectional study. Our outcome of interest was self-reported and may be subject to recall bias. However, we do not expect recall to be different between mothers who had early ANC and mothers who did not. Similarly, planned pregnancy status was self-reported and we expect recall bias to be similar between mothers who received IPTp+3 and those who did not. Another limitation was that we adjusted for a limited number of confounding variables, and therefore there is potential for residual confounding.

## Conclusions

Early ANC is likely to be a major determinant towards meeting the WHO 2012 guidelines on IPTp+3, and therefore its initiation should be prioritized, particularly among women whose pregnancies are unplanned. Further studies with a cohort design are needed to replicate our effect modification analyzes in this population.

## Data Accessibility Statement

Data are publicly available from: *https://mics.unicef.org/surveys*.

## Additional File

The additional file for this article can be found as follows:

10.5334/aogh.3550.s1 Supplementary Files. Supplementary Figures and Tables.
