# Supplementary material for: The Association Between Early Antenatal Care and Intermittent Preventive Treatment of Malaria in Pregnancy in Sub-Saharan Africa: Effect Modification by Planned Pregnancy Status
Source: Ann Glob Health. 2022 Jan 10;88(1):4. doi: 10.5334/aogh.3550 (PMC8757383; doi:10.5334/aogh.3550)
Supplement: Supplementary Files. — Supplementary Figures and Tables. [file agh-88-1-3550-s1.pdf]

Supplementary Files.

Supplementary Figure S1. Flow diagram showing screening of MICSs for study inclusion.

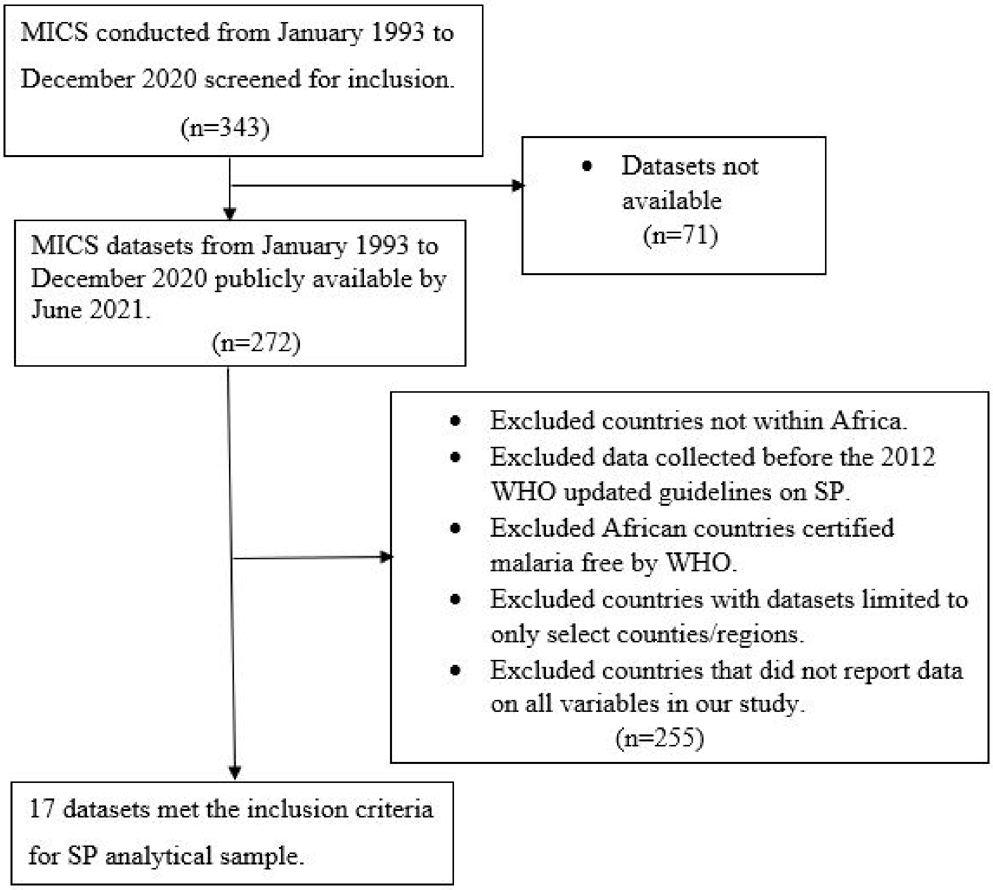

Supplementary Table S1. Coverage of intermittent preventive treatment by country

**Country**	IPTp1+ (95% CI)	IPTp2+ (95% CI)	IPTp3+ (95% CI)
Benin	64.2% (62.2%, 66.2%)	37.6% (35.6%, 39.7%)	12.5% (11.2%, 13.9%)
CAR	59.7% (57%, 62.4%)	46.3% (43.7%, 49%)	30.2% (28.1%, 32.4%)
Cameroon	75.5% (73.6%, 77.5%)	53.2% (50.6%, 55.7%)	26.1% (23.9%, 28.3%)
Congo	43.5% (40.5%, 46.6%)	24.7% (22.2%, 27.2%)	12% (10.5%, 13.6%)
DRC	56% (52.7%, 59.4%)	31.2% (27.3%, 35.1%)	13.4% (9.9%, 16.8%)
Gambia	97.4% (96.6%, 98.2%)	75.2% (73.4%, 77.1%)	37.5% (35.2%, 39.9%)
Ghana	92.1% (90.8%, 93.3%)	77.1% (74.9%, 79.3%)	51.7% (49.2%, 54.1%)
Guinea	62.2% (59.5%, 65%)	48.9% (46.3%, 51.5%)	30% (27.6%, 32.3%)
Guinea Bissau	81.4% (79.3%, 83.5%)	60% (56.9%, 63.1%)	24.2% (21.6%, 26.8%)
Ivory Coast	66.6% (63.6%, 69.5%)	46.4% (43.7%, 49.2%)	22.5% (20.4%, 24.5%)
Madagascar	42.9% (40.4%, 45.3%)	28.6% (26.5%, 30.8%)	14.9% (13.4%, 16.3%)
Malawi	89.8% (88.7%, 91%)	59% (57.4%, 60.7%)	19.4% (18.1%, 20.7%)
Mali	64.6% (62%, 67.3%)	40.2% (37.9%, 42.4%)	16.2% (14.6%, 17.8%)
Mauritania	54.7% (52%, 57.4%)	28.8% (26.5%, 31.1%)	10.6% (8.9%, 12.3%)
Nigeria	47.9% (42.2%, 53.6%)	31.2% (27.9%, 34.5%)	14.7% (13.2%, 16.2%)
STP	94.1% (92.2%, 95.9%)	10.8% (7.9%, 13.7%)	2.9% (1.3%, 4.4%)
Togo	89.1% (87.2%, 91.1%)	64.7% (61.8%, 67.6%)	37.1% (34.3%, 40%)

CAR: Central African Republic; DRC: Democratic Republic of the Congo; STP: São Tomé and Príncipe
IPTp3+: three or more intermittent preventive treatment doses
IPTp2+: two or more intermittent preventive treatment doses
IPTp1+: one or more intermittent preventive treatment doses

Supplementary Table S2. Effect modification of the association between early antenatal care and IPTp2+ by planned pregnancy status

	No early ANC		Early ANC		
	N with/without outcome	PR (95% CI)	N with/without outcome	PR (95% CI)	PR (95% CI) comparing IPTp2+ coverage with early ANC vs. not within strata of planned pregnancy status
Planned pregnancy	11633/17207	1.00 (Reference)	9385/9469	1.12 (1.08,1.16) p*<0.01*	1.12 (1.08,1.16) p*<0.01*
Unplanned pregnancy	5755/8943	0.89 (0.86,0.93) *p<0.01*	4039/4443	1.09 (1.05,1.13) *p<0.01*	1.22 (1.17,1.27) p<0.01

Measure of effect modification on additive scale: RERI (95% CI) = 0.07 (0.02, 0.12); p<0.01
Measure of effect modification on multiplicative scale: ratio of PRs (95% CI) = 1.09 (1.03, 1.15); p<0.01
Prevalence ratios (PRs) are adjusted for age, marital status, education, household wealth, place of residence, parity, access to media, perceived domestic violence
IPTp2+: two or more intermittent preventive treatment doses

Supplementary Table S3. Effect modification of the association between early antenatal care and IPTp1+ by planned pregnancy status.

	No early ANC		Early ANC		
	N with/without outcome	PR (95% CI)	N with/without outcome	PR (95% CI)	PR (95% CI) comparing IPTp1+ coverage with early ANC vs. not within strata of planned pregnancy status
Planned pregnancy	17765/11075	1.00 (Reference)	13066/5790	1.04 (1.02,1.06) p*<0.01*	1.04 (1.02,1.06) p*<0.01*
Unplanned pregnancy	9249/5450	0.95 (0.93,0.97) *p<0.01*	5982/2500	1.03 (1.01,1.06) *p<0.01*	1.09 (1.06,1.11) p<0.01

Measure of effect modification on additive scale: RERI (95% CI) = 0.04 (0.0007, 0.08); p=0.04
Measure of effect modification on multiplicative scale: ratio of PRs (95% CI) = 1.04 (1.01, 1.08); p=0.01
Prevalence ratios (PRs) are adjusted for age, marital status, education, household wealth, place of residence, parity, access to media, perceived domestic violence
IPTp1+: one or more intermittent preventive treatment doses
